# A linear polyethylenimine mediated siRNA-based therapy targeting human epidermal growth factor receptor in SPC-A1 xenograft mice

**DOI:** 10.1186/2213-0802-1-2

**Published:** 2013-02-22

**Authors:** Pinghai Zhang, Nuo Xu, Lei Zhou, Xin Xu, Yuehong Wang, Ka Li, Zhaochong Zeng, Xiangdong Wang, Xin Zhang, Chunxue Bai

**Affiliations:** 1 Department of Pulmonary Medicine, Zhongshan Hospital, Fudan University, Shanghai, 20032 China; 2 Department of Radiotherapy, Zhongshan Hospital, Fudan University, Shanghai, 20032 China

**Keywords:** RNA interference, Small interfering RNA, Epidermal growth factor receptor, Linear-polyethylenimine, Non small cell lung cancer, Intraperitoneal injection

## Abstract

**Background:**

Linear polyethylenimine (LPEI) is considered as a desirable gene *in vivo* delivery system, but whether it could deliver the specific siRNA targeted EGFR to the tumor site to inhibit the growth of NSCLC xenograft in nude mice still needs to be examined.

**Methods:**

In this study, LPEI/siRNA was made into a complex and SPC-A1-xenografted mice model was established. Then, stable LPEI/siRNA-EGFR complexes were intraperitoneally administrated. Afterwards, tumor growth was measured every 3 days. At the end of the experiment, tumor volume was calculated, and tumors were weighed, and examined for EGFR expression, proliferation, and apoptosis evaluations. By using blood samples, toxicity tests including aspartate aminotransferase (AST), alanine aminotransferase (ALT), urea and creatinine (Cr) were measured for liver and renal function evaluation. Serum concentrations of TNF-α and IFN-γ were also examined.

**Results:**

Our results demonstrated that LPEI/siRNA-EGFR complexes could downregulate EGFR expression in SPC-A1 xenografted tumor upon single i.p. injection. LPEI/siRNA-EGFR complexes inhibited tumor growth and did not induce organ toxicity in SPC-A1-xenografted mice. At the end of the experiment no significant IFN-α increase was detected in LPEI/siRNA complexes or glucose-treated groups.

**Conclusions:**

The novel modality of siRNA-based therapy targeting EGFR may be of great potential in NSCLC treatment.

**Electronic supplementary material:**

The online version of this article (doi:10.1186/2213-0802-1-2) contains supplementary material, which is available to authorized users.

## Background

Non-small-cell lung cancer (NSCLC) is a major global health problem and is the leading cause of cancer death worldwide [1]. Currently, non-specific and non-selective treatment with cytotoxic chemotherapy results in only a modest increase in survival, but at the cost of significant toxicity. This underscores the need for novel agents with improved efficacy and safety profiles [2–5]. Among those, the epidermal growth factor receptor (EGFR) is one of the most important targets in NSCLC therapy [6]. The currently available agents targeting EGFR in NSCLC treatment are two small molecular tyrosine kinase inhibitors (TKIs), including gefitinib and erlotinib. A phase III, open-label study demonstrated the presence in the tumor of a mutation of the EGFR gene is a strong predictor of a better outcome with gefitinib [7]. Even more, EGFR-TKI was recently recommended to be the first-line therapy in EGFR-mutation positive patients with NSCLC. Unfortunately, the responsive rate to the EGFR-TKIs is low for reasons such as primary or secondary resistance [8–12]. Thus, no adequate treatment options currently exist for EGFR-tumor-driven patients who have experienced EGFR-TKIs and chemotherapy failure [13].

In recent years, RNA interference (RNAi) has drawn attention in therapeutic studies of cancer [14–16]. It was discovered to be a process of post-transcriptional gene silencing, which induces degradation of a homologous messenger RNA (mRNA) and protein knock-down in a sequence-specific manner [17, 18]. Due to this property, siRNA sequences have been designed for many target mRNAs. For example, the target of interest could be a mutated protein, as in the case of NSCLC patients resistant to EGFR-TKIs. Additionally, conventional small molecule drug discovery involves complicated screening and random modifications, thus leading to new compounds. However, to design a therapeutic siRNA requires only knowledge of the target gene’s sequence, and thus can be chemically synthesized at relatively low cost. In this regard, the novel therapeutic modality of siRNA-based gene silencing may have advantages over drugs currently on the market.

However, for further *in vivo* application, the negative charge and chemical degradability of siRNA under physiologically relevant conditions make its delivery a major challenge. Viral vectors have high *in vitro* gene transfer efficiency, but their limited carrying ability of genetic material and severe safety risks induced by their immunogenicity and oncogenic potentials deter broad use in humans [19]. Recently, non-viral vectors have been investigated for their ease of preparation, purification, and chemical modification, as well as their stability. Among these lipoplexes have shown significant toxicities. In contrast, the cationic polymers, polyethylenimine (PEIs), are regarded as the most efficient and versatile non-viral vectors [20]. Of these, linear PEI (LPEI) has been widely employed in gene transfer into varied organs [21], such as lung, brain, retina, pancreas, and bladder tumors [22–25]. Clinical trials for the treatment of bladder cancer and human immunodeficiency virus (HIV) infection have been underway, using LPEI-mediated plasmid DNA delivery [26]. Moreover, this delivery system has recently been successfully applied in siRNA delivery *in vivo* [27]. The positive charges of LPEI are felt to allow efficient interaction with siRNA, forming complexes which bind onto cell membranes and undergo endocytosis. Then, LPEI uses the so-called “proton sponge” effect to enhance endosomal release of the polyplexes and bringing out the RNAi to plasma [28].

Based on the above, The ultimate aim of our study was to discover novel siRNA-based therapy targeting EGFR in human NSCLC. First, we tested whether LPEI could efficiently deliver EGFR-specific siRNA to the tumor site, leading to an antitumor effect in human NSCLC cell xenografts, when administered by intraperitoneal injection. Then toxicity and immunogenic reactions after systemic release also evaluated for safety.

## Methods

### Cell lines and animals

SPC-A1, a well-characterized human lung adenocarcinoma cell (HLAC) line, was obtained from Shanghai Cell and Biology Institute. Cells were maintained in RPMI 1640 medium (Gibco, USA) supplemented with 10% bovine serum (Gibco, USA), 2mmol/L L-glutamine and antibiotics (100U/mL penicillin and 100 μg/mL streptomycin) in a humidified atmosphere of 5% CO_2_ at 37. The 8-week-old male BALB/C nude and immunocompetent mice (body weight 20±2g) bought from Chinese Academy of Science, were kept in filter-topped cages with standard rodent chow and water available *ad libitum*, and a 12 hours light/dark cycle. Those nude mice were randomly allocated into different groups and there were 6 mice for each group. All animal protocols were approved by the Ethical Committee on Animal Experiments of the University of Fudan Animal Care Committee, Shanghai, China. All efforts were made to minimize suffering.

### siRNAs

Human EGFR (NM_005228) gene targeted siRNA-EGFR (5′-GGAGCUGCCC AUGAGAAAUdTdT-3′/5′-AUUUCUCAUGGGCAGCUCCdTdT-3′) and non-specific siRNA-NEG (5′-GGAGCUGCCCAUGAGAAAUdTdT-3′/5′-AUUUCUCAUGGGCAG CUCCdTdT-3′), designed as previous description [29], were chemically synthesized and annealed by GenePharma (Shanghai, China). Aqueous siRNA (20 μmol/L and 200 μmol/L) were subpackaged and stored at −80.

### LPEI/siRNA complexes preparation and cell transfection in vitro

Commercial low molecular weight (22 kDa) Linear-PEI (*jet* PEI^TM^), for *in vitro* DNA transfection, was bought from PolyPlus-transfection (Illkirch, France) and stored at −80. LPEI consisted of 7.5 mmol/L of NH_4_^+^. LPEI/siRNA was made into a complex. In brief, 5 μl siRNA was dissolved in 100 μl of 10 mmol/L HEPES-150 mmol/L NaCl, pH 7.4, and incubated for 10 minutes. LPEI (2.5 μl) was dissolved in 100 μl of the same buffer and, after 10 minutes, was pipetted into the siRNA solution. This gave a net molar excess of ionizable nitrogen of LPEI to phosphate of siRNA (N/P) at a ratio of 5 as suggested for DNA by the manufacturer. Corresponding LPEI/siRNA complexes were constructed at N/P ratios of 3, 5 and 10. The total 200 μl mixture was vortexed and incubated at room temperature for 20 minutes to form a stable LPEI/siRNA-EGFR complex, and then dropped slowly into each well of 6-well plates. Serum-free DMEM was supplemented to a final volume of 2 mL, and 4 hours later replaced with RPMI 1640 medium.

Cells were harvested 24 hours after transfection. Total RNA was isolated from cells using TRIZOL Reagent (Invitrogen, USA), purified as recommended by the manufacturer, and then reverse-transcribed with M-MLV Reverse Transcriptase Kit (Promega, USA) following the manufacturer’s instructions. Real-time RT-PCR was performed using SYBR Green Real time PCR Master Mix (TOYOBO, Japan) and amplified with a Roter-Gene 3000 Amplification System (Corbett Research, Australia). Primer sequences used for EGFR (forward 5′-ACCGTGCCCTGATGGATGA-3′, reverse 5′-CCACGGTGGAATTGTT GCTG-3′) and β-actin (forward 5′-ATGACCCAGATCATGTTTGAGACC-3′, reverse 5′-GGAGGGCATACCCCTCGTAGA-3′) were designed and synthesized by Sangon Co. (Shanghai). All samples’ EGFR mRNA expression levels were normalized by β-actin amplification.

Flow cytometry assays were performed at 48 hours after transfection for the EGFR numbers scoring. After trypsinizing from plates, cells were washed twice with 1×PBS, then incubated with 20 μl of R-PE-conjugated mouse anti-human EGFR monoclonal antibody (BD Biosciences, USA) for 15 min in the dark at room temperature. Afterwards, cells received the same washing process and fixed in 0.5 mL of 4% paraformaldehyde. The EGFR numbers were analyzed on a Becton Dickinson FACScan flow cytometry with excitation and emission settings of 488 nm and 575 nm, respectively. To determine the change of EGFR numbers in SPC-A1 cells, we used positive cell percentage × mean intensity to evaluate the intensity of fluorescence.

For specificity evaluation, non-specific siRNA-NEG was complexed by LPEI at corresponding N/P ratios and thereafter transfered into SPC-A1 cells. Lipofectamine 2000 (Invitrogen, Carlsbad, CA, USA) complexed siRNA-EGFR transfecting group was set as a positive control. The non-treatment group was taken as a blank control. EGFR mRNA or protein expression levels were normalized by setting the amount of blank control at 1.0.

### Size and zeta-potential measurements

Dynamic light scattering (DLS) was employed to determine hydrodynamic diameters of the complexes, and laser Doppler anemometry (LDA) was utilized to measure their zeta potentials. LPEI complexed siRNA nanoplex was prepared as above. Plasmid DNA (*p* ShNEG) provided by p*Silencer*^TM^ 2.1-U6 neo kit (Ambion, Inc., Austin, TX, USA) was complexed with LPEI at the same N/P ratio of 5 as the manufacturer’s instructions. When the LPEI/siRNA and LPEI/DNA complexes were formed, their mean particle size and zeta-potential distribution were measured using a Zeta potential/particle Sizer Nicomp^TM^ 380 ZLS (PSS Nicomp particle size system, USA). Position and attenuator were optimized by the device. Measurements were conducted in quadruplicates.

### LPEI/siRNA complexes transfection in vivo by intraperitoneal (i.p.)injection

SPC-A1 cell suspension was prepared and inoculated subcutaneously into flanks of nude mice (1×10^7^ cells /100 μl per site), (n = 6). As grown up tumors were removed and divided into uniform masses of 1 mm × 1 mm, and implanted subcutaneously into the right flank of the untreated mice. When tumors reached 6 mm × 6 mm, the SPC-A1-xenografted mice model was successfully established and i.p. treatment started.

For i.p. injection, the in vivo-*jet* PEI^TM^ transfection reagent (optimized cationic linear PEI-based transfection reagent for *in vivo* experiments), also from Polyplus-transfection (Illkirch, France), was used, which provided as a ready-to-use solution at 150 mM nitrogen concentration and less than 0.1EU/mL endotoxin. SiRNA-EGFR (3 μl, 200 μmol/L) and LPEI (0.75μl) were dissolved in 250μl of 5% glucose solution (GS), respectively. The latter was pipetted into the siRNA solution 10 minutes later. After vortexing and incubating at room temperature for 20 minutes, stable LPEI/siRNA-EGFR complexes under N/P ratio of 5 were formed in 5% GS at a final volume of 500μl. Afterwards, they were intraperitoneally administrated to each mouse in 1h. At the same time, a corresponding dose of LPEI complexed non-specific siRNA-NEG or 5% GS were given to the other two groups as controls.

In the experiment of discontinued administration, i.p.injection was started (day 0) when tumors had reached 6 mm × 6 mm, and repeated 2~3 times per week until the 22nd day, 24 h after the last injection. Tumor growth was measured every 3 days using a digital caliper by an observer blinded to treatment allocation. Tumor volume was calculated (0.52× longest diameter × shortest diameter^2^) [30]. At the end of the experiment, tumors were harvested, weighed, and examined for EGFR expression, proliferation, and apoptosis evaluations. No deaths of nude mice was observed during the experiment process.

### Western blot analysis

EGFR expression in tumor samples was detected by western blot. Tissue was homogenized, centrifuged, and supernatants collected. Equivalent amounts of extracted protein (50 mg) were mixed with sample buffer containing 5% 2-mercaptoethanol, boiled, cooled, and loaded in each lane of a 7.5% polyacrylamide gel. Electrophoresis was performed at a constant voltage of 80V and, subsequently, proteins were transferred to a PVDF membrane. The membrane was blocked overnight with 3% gelatin in Tris-buffered saline (TBS). Subsequently, membranes were incubated with mouse anti-EGFR primary antibody (1:1000, BD Biosciences, USA) at 4°C overnight, and after washing twice in TBST, with peroxidase-conjugated goat anti-mouse IgG (Santa Cruz biotechnology, Inc., Santa Cruz, CA, USA) at room temperature for 1 hour. GAPDH was used as an internal control. Protein blots were visualized with chemiluminescence reagent ECL(Amersham, Freiburg, Germany). Membranes were washed thrice and then exposed to X-ray film and bands were quantified by scanning densitometry.

### Immunohistochemisty and terminal deoxynucleotidyl Transferase Biotin-dUTP Nick End Labelling (TUNEL) assay

Tumors were fixed in 4% paraformaldehyde and embedded in paraffin. IHC detection of EGFR and proliferating cell nuclear antigen (PCNA) were performed in 3μm histological sections. Briefly, sections were immersed in xylene, 95% alcohol, and 80% alcohol for 10 min, respectively, and washed with PBS (pH 7.4) for three times after each immersion. After protein denature, using microwave and non-specific biding blocking with normal goat serum for 20 min at RT, sections were incubated with primary antibodies against EGFR or PCNA (both diluted to 1:100, Maixin-Bio, China) overnight at 4°C. Sections were washed with PBS and incubated with goat anti-mouse secondary antibody (Cell Signaling) at a 1:100 dilution for 20 min at 37°C. Sections were again washed with PBS and incubated for 20 min with SABC. After again being washed with PBS, sections were incubated with 3, 3-di-aminobenzidine (DAB) for 3~5 min, and the reaction stopped by washing in PBS. Microscopically, brown particles appeared within cells, indicating positive expression of the protein molecules assessed. Five consecutive high-power fields (×200) were examined in each of specimen under a light microscope (Carl Zeiss, Germany) in a blinded fashion. The proliferation index was calculated according to the following formula: number of PCNA positive cells/ total cell count × 100%.

LPEI/siRNA complexes inducing cell apoptosis was assessed by measuring DNA strand breaks using an *in situ* cell death detection kit (Roche Applied Science, Indianapolis, IN, USA) based on TUNEL staining. Tissue slides were fixed in 4% paraformaldehyde for 10 minutes, followed by washing in phosphate buffered saline (PBS) and blocking in 3% H_2_O_2_ methanol for 10 min. After permeabilisation in a solution containing 0.1% Triton X-100 and 0.1% sodium citrate, tissues were labelled with 25 ml of TUNEL reaction mixture containing a 1:2 dilution of enzyme for 2 hours at 37°C in a humidified chamber. Signals were then converted into HRP using antifluorescein antibody and visualized by 3,3′diaminobenzidine coloration (Roche Applied Science, Indianapolis, Indiana, USA) as recommended by the manufacturer. Tissues were counterstained with methylgreen and TUNEL-positive cells were counted in five randomly selected × 200 high-power fields under microscopy. The apoptosis index was calculated according to the following formula: number of apoptotic cells/total number of nucleated cells × 100%.

### Blood biochemical analysis

At the endpoint of the above experiment, all blood samples were collected for toxicity testing. By retro-orbital puncture, blood was collected into heparinized tubes. Within 1 h, samples were centrifuged at 5000 × g for 10 min. The plasma was taken for biochemical parameters analysis, carried out with an automatic analyzer (HITACHI 7020, JAPAN). Liver enzymes, including aspartate aminotransferase (AST), and alanine aminotransferase (ALT), were measured. Urea and creatinine (Cr) were also measured for renal function evaluation.

### Immunogenicitiy assay

SiRNA-EGFR or non-specific siRNA-NEG (0.6 nmol), both complexed with LPEI in 5% glucose solution, were administrated by i.p. injection to 8-week-old male BALB/C nude or immunocompetent mouse, and 500 μl of 5% glucose solution was given as a negative control. After 2 days, the injection was repeated at 6 h before the end of the experiment. As a positive control based on a previous study, 40 μg of lipopolysaccharide (LPS, Sigma, St. Louis, MO) in 500 μl of phosphate-buffered saline (PBS) was intraperitoneally administrated to immunocompetent mouse and the experiment terminated after 2 h. Mice blood was taken by retro-orbital puncture and allowed to clot overnight at 4°C. Samples were centrifuged at 5000 × g for 10 min, and the supernatants collected. Serum concentrations of TNF-α and IFN-γ were determined by enzyme-linked immunosorbent assay (ELISA) kits according to manufacturer’s instructions (R&D Systems, Minneapolis, MN, USA). The amount of cytokine was determined on 100 μl of × 5 diluted serum, loaded in duplicate.

### Statistical analysis

Data were presented as the means ± standard deviation. Statistical differences among multiple groups were calculated by one-way analysis of variance (ANOVA). If a ANOVA was statistically significant, an unpaired two-tailed Student’s *t*-test was used for between-group comparisons. *P* values of less than 0.05 were considered statistically significant.

## Results

### In vitro optimizing of LPEI/siRNA-EGFR complexes

**Figure 1:**
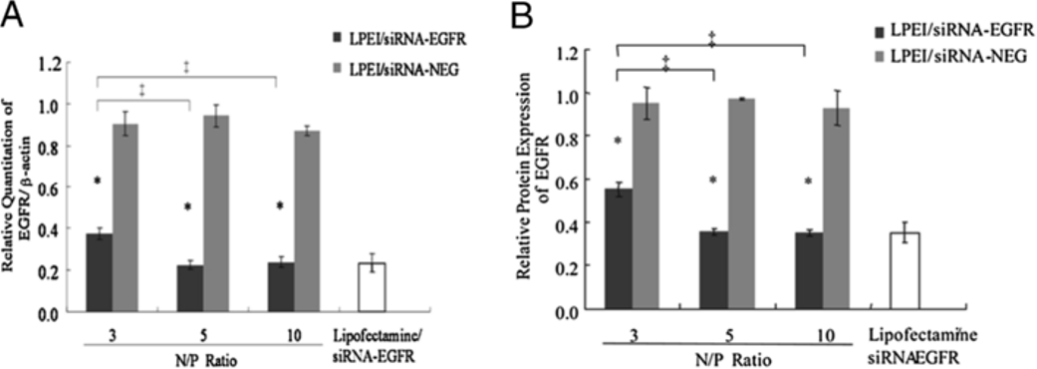
***In vitro*** **comparison of transfection efficiencies.** *In vitro* comparison of transfection efficiencies of LPEI/siRNA-EGFR under various N/P ratios (black panels), LPEI/siRNA-NEG (grey panels), and Lipofectamine/siRNA-EGFR (white panel) transfected groups. SPC-A1 cells were seeded into 6-well plates at 3 × 105 cells/well and, at ~50% confluency, transfection was performed as indicated. Cells were harvested **(A)** after 24 hours for quantitative expression of EGFR mRNA by real-time RT-PCR analysis, **(B)** and after 48 hours for EGFR protein by flow cytometry assays. Data are expressed as means±s.d. of numbers obtained from 6 animals in each group. All the results are shown as the mean±standard error of the mean (SEM). * P<0.05 when compared with respective LPEI/siRNA-NEG groups. ‡ P<0.05 when compared with LPEI/siRNA-EGFR group at different N/P ratios.

We first performed an *in vitro* experiment to test whether LPEI could deliver unmodified siRNA efficiently into human lung cancer cells and exert a specific gene silencing effect. The N/P ratio, which indicates quotient of the nitrogen atoms of LPEI to siRNA phosphates in the complexes, will determine particle size and zeta-potential, thus influencing the efficiency of siRNA delivery. LPEI/siRNA-EGFR complexes were prepared at various N/P ratios (= 3, 5, 10, respectively) and their silencing efficiencies were evaluated in the SPC-A1 cell line, previously proven to have a high level of EGFR expression [31]. Compared to non-specific LEPI/siRNA-NEG groups, treatment with a single dose (100pmol) of LPEI-complexed siRNA-EGFR resulted in target gene expression down-regulation under each N/P ratio, with all differences being significant. Under an N/P ratio of 5, 76.42% of down-regulation in EGFR mRNA was obtained at 24h after LPEI/siRNA-EGFR transfection (Figure 1A). An EGFR protein reduction of 63.53% was also confirmed by flow cytometry assays at 48h following treatment (Figure 1B). These results were similar to those of the Lipofectamine/siRNA-EGFR -transfected group. The lower efficiency at N/P ratio of 3 may have been due to an insufficient complexation of siRNA by LPEI (data not shown). Additionally, because the dose increase of LPEI under N/P ratio of 10 did not lead to a higher silencing efficacy, the optimal N/P ratio was considered as 5 for LPEI-vectored siRNA-EGFR delivery during the *in vitro* and further *in vivo* studies.

To explain the optimal silencing efficiency produced by LPEI-complexed siRNA, the mean particle size and zeta-potential distribution were determined. Furthermore, considering that *jet* PEI was introduced previously as a DNA plasmid transfection reagent, these parameters were compared with LPEI/plasmid DNA complexes using the same N/P ratio. As displayed in Table 1, the electrostatic interactions between LPEI and siRNA resulted in formation of complexes with average particle size distribution of ~100 nm, while LPEI/DNA complexes showed ~2-fold increase in size, revealing that LPEI/siRNA complexes were more compact as compared with LPEI/DNA complexes. Similar lower positive surface potential were detected in both complexes.

**Table 1:** **Characteristics of LPEI-complexed nucleic acid under N/P ratio of 5**

Complexes type	Mean particle size (nm)	Zeta-potential (mV)
LPEI/siRNA	87.13±11.11	6.22±1.63
LPEI/DNA	148.93±9.71	7.44±0.58

All data were presented as means ± s.d of numbers obtained from 6 animals in each group.

### LPEI/siRNA-EGFR complexes downregulate EGFR expression in SPC-A1 xenografted tumor upon single i.p. injection

**Figure 2:**
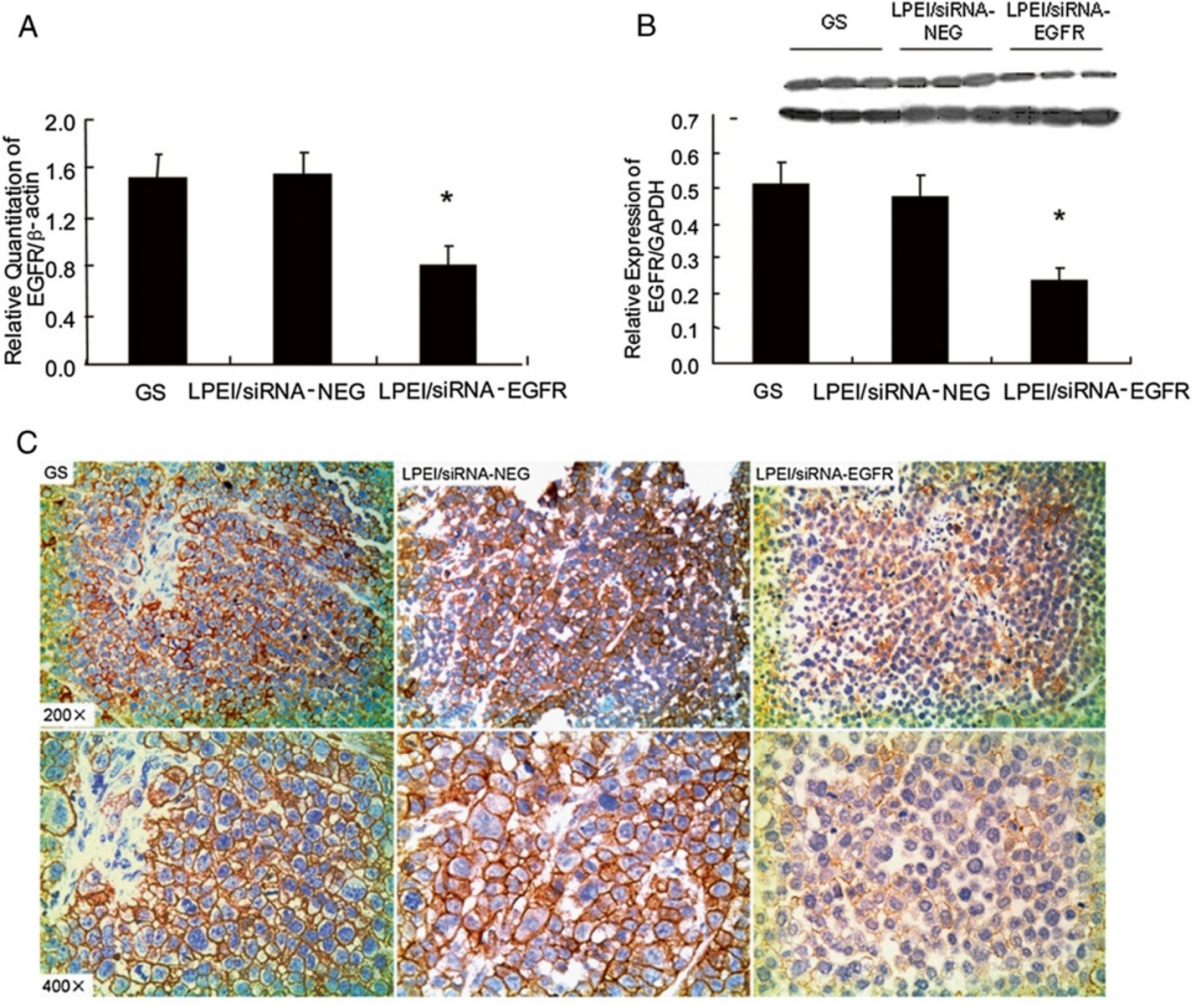
**Reduction of EGFR expression in SPC-A1 xenografts upon LPEI/siRNA-EGFR complexes injection, compared with control groups.** **(A)** EGFR mRNA quantitative evaluation by real-time RT-PCR at 24 hours after injection. **(B)** Western Blot analysis, and **(C)** IHC staining of EGFR protein determination at 48 hours after injection. EGFR seen as brown stain, with magnification ×200 (upper) and ×400 (lower)(n = 6).All the results are shown as the mean±standard error of numbers obtained from 6 animals in each group. *P < 0.05.

The ultimate goal of our study was to explore the therapeutic use of EGFR-specific siRNA through systemic application. Indeed, it has been known that many vectors have failed with *in vivo* gene delivery in spite of good transfection abilities *in vitro*. Therefore, we examined whether LPEI-complexed siRNA-EGFR under N/P ratio of 5 could also induce efficient target gene silencing upon a single i.p. injection *in vivo*. SPC-A1-xenografted mice were randomly divided into three groups. As tumors reached 6 mm×6 mm, each mouse was intraperitoneally injected with 0.6 nmol of siRNA-EGFR complexed with LPEI. Corresponding doses of LPEI/siRNA-NEG or 5% GS were administrated as control groups. Twenty-four hours later, the mice were killed and their tumors processed for RNA extraction. Quantitative real-time PCR analysis (Figure 2A) revealed a 49% reduction of EGFR mRNA content of tumors treated with LPEI/siRNA-EGFR complexes in comparison with the non-specific LPEI/siRNA-NEG control, the results showing no significant difference with GS control group. Consistently, at 48h after LPEI/siRNA-EGFR complexes injection, tumor western blot analysis (Figure 2B) showed significant decrease (51%) in EGFR protein expression as compared with the non-specific control group. IHC examination (Figure 2C) confirmed a marked EGFR silencing efficiency of the LPEI/siRNA-EGFR complexes. These results suggested that EGFR-specific siRNA complexed with LPEI could efficiently reduce EGFR expression at both mRNA and protein levels in SPC-A1-xenografted tumors, with no non-specific RNAi effect being observed.

### LPEI/siRNA-EGFR complexes inhibit tumor growth following repeated i.p. administration

**Figure 3:**
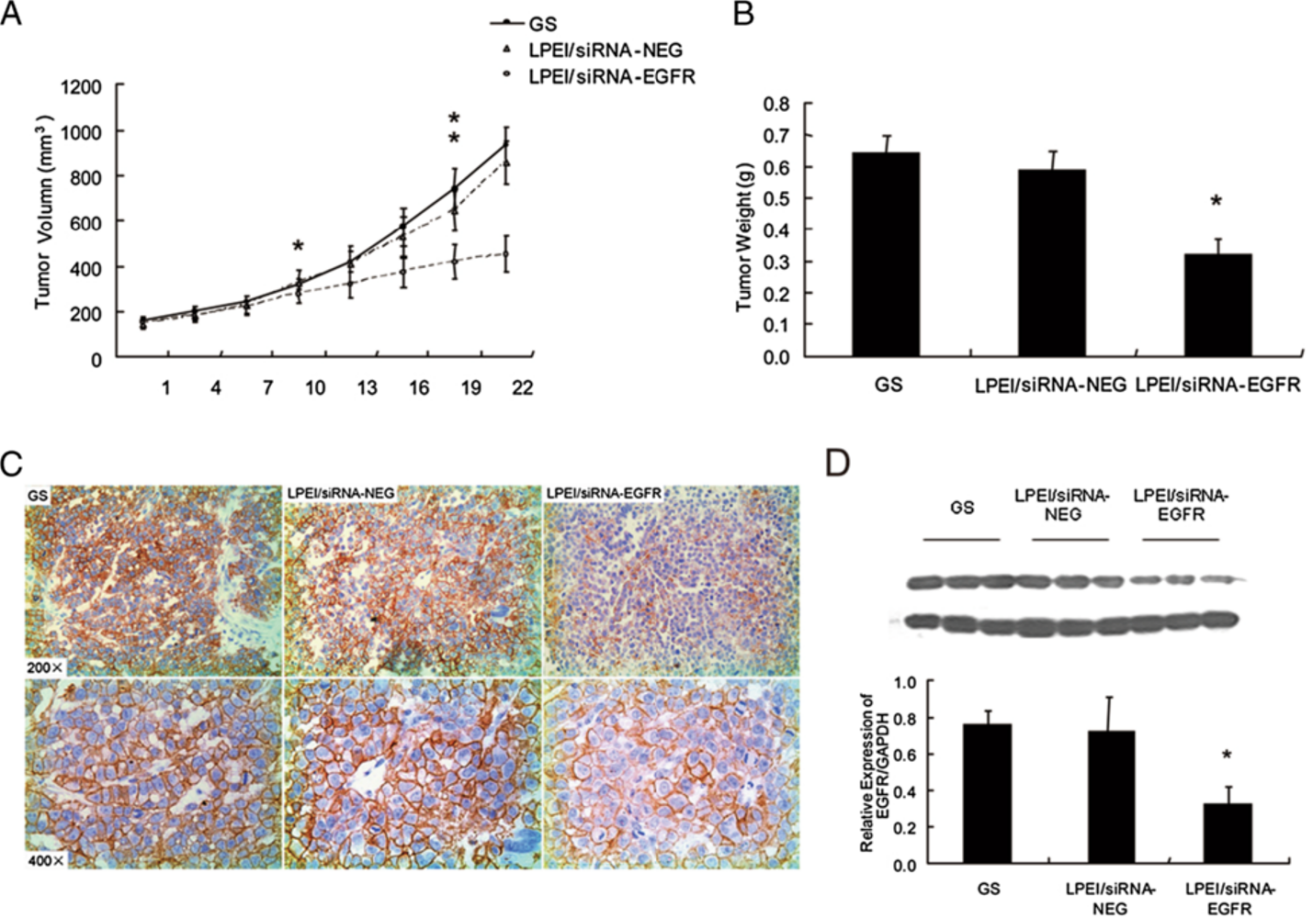
**Growth inhibition of SPC-A1 xenografts.** Followed by systemic treatment with LPEI-complexed EGFR-specific siRNA, the growth of SPC-A1 xenografts has been inhibited because of decreased EGFR expression. Subcutaneous xenografts generated as described above. **(A)** Intraperitoneal injection performed every 3 days with 0.6 nmol LPEI/siRNA-EGFR (open circles), LPEI/siRNA-NEG (closed circles), and 5% GS (triangles), respectively. Tumor volumes recorded every 3 days. Data expressed as mean of six mice in each group. **(B)** Tumor weight comparisons between three groups at endpoint of experiment. **(C)** IHC and **(D)** Western blot analysis for EGFR protein levels. All the results are shown as the mean±standard error of numbers obtained from 6 animals in each group. *P < 0.05; **P < 0.01.

Previously we demonstrated that stable downregulation of EGFR expression by vector-based shRNA significantly inhibited NSCLC cells proliferation [31]. Therefore, we used the athymic nude mouse model to test if repeated systemic administration of LPEI-complexed and EGFR-specific siRNA could result in growth inhibition of SPC-A1-xenografted tumors. Mice were grouped and treated as described above. As shown in Figure 3A, tumors in mice treated with LPEI-complexed non-specific siRNA grew similar to those of GS-treated mice and followed for 2 weeks. Nonetheless, there was no significance difference. However, treatment with EGFR-specific siRNA vectored by LPEI induced significant tumor growth inhibition. Differences reached statistical significance at day 10, and tumor weight reduction at day 22 was almost 50% (Figure 3B). Also, tumor IHC revealed a marked downregulation of EGFR protein expression in the LPEI/siRNA-EGFR-treated group (Figure 3C), and, as shown by western blot analysis, inhibitory efficiency was 55% (Figure 3D).

**Figure 4:**
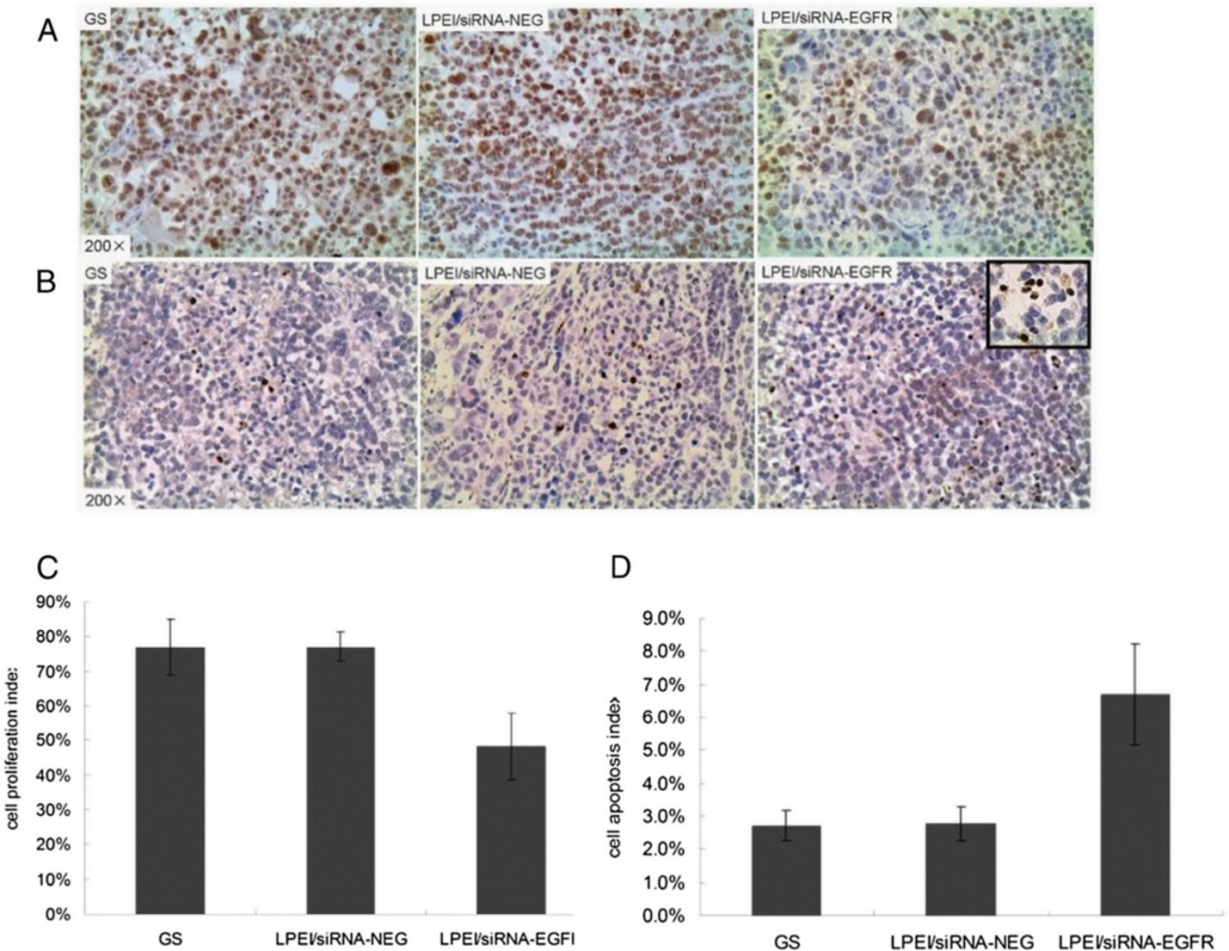
**After repeated i.p. administration, tumor PCNA protein expression detected by IHC.** **(A)** and apoptosis examined by TUNEL assay **(B)**. Quantitative analysis data revealed 37% reduce in cell proliferation index in LPEI/siRNA-EGFR group compared with other two groups **(C)**, and more than twofold increase in apoptosis index under LPEI /siRNA-EGFR treatment **(D)**. All the results are shown as the mean±standard error of numbers obtained from 6 animals in each group. *P < 0.05.

To identify tumor proliferation and apoptosis following i.p. injections with LPEI-complexed EGFR specific siRNA, we also examined PCNA expression and apoptosis-induced DNA fragmentation in mice xenografts. As shown (Figure 4A), in the LPEI/siRNA-EGFR-treated group, PCNA positive staining was reduced, while TUNEL-positive cell counts were significantly higher than in the other two groups (Figure 4B). Comparisons between the three groups revealed a ~30% descent in the proliferation index (Figure 4C), as well as a >twofold increment in the apoptosis index (Figure 4D) under LPEI-complexed EGFR-specific siRNA treatment. In contrast, no significant difference was found in cell proliferation or apoptosis between LPEI/siRNA-NEG and GS treated groups. These results provide evidence that the observed inhibitory effect on tumor growth was based on specific EGFR down-regulation, acting through modulations of cell proliferation and apoptosis.

### Toxicity

To determine whether following repeated systemic administration with the LPEI/siRNA-EGFR complexes there could be organ toxicity, mice blood levels of ALT and AST, urea and creatinine (Cr) were measured. As shown in Table 2, there was no evidence of increased liver toxicity or significance renal toxicity. In addition, microscopically, there was neither necrosis nor inflammatory cell infiltration of the heart, liver, lungs, or kidneys (data not shown). These results suggest that the therapy of repeated i.p. administration with 0.6 nmol EGFR-specific siRNA vectored by LPEI does not induce organ toxicity in SPC-A1-xenografted mice.

**Table 2:** **Liver and renal function evaluation following repeated i.p. injection in tumor-xenografted mice**

Group	ALT (IU/L)	AST(IU/L)	Urea (mmol/L)	Cr (μmol/L)
5% GS	58.33±11.38	188.17±39.75	9.73±0.97	48.43±2.48
LPEI/siRNA-NEG	55.00±11.03	184.67±62.24	10.45±0.68	47.70±3.61
LPEI/siRNA-EGFR	53.83±13.47	178.33±53.41	10.35±1.04	46.87±7.55

Data presented as means±s.d. of numbers obtained from 6 animals in each group.

### Immunogenicity of LPEI/siRNA-EGFR

**Figure 5:**
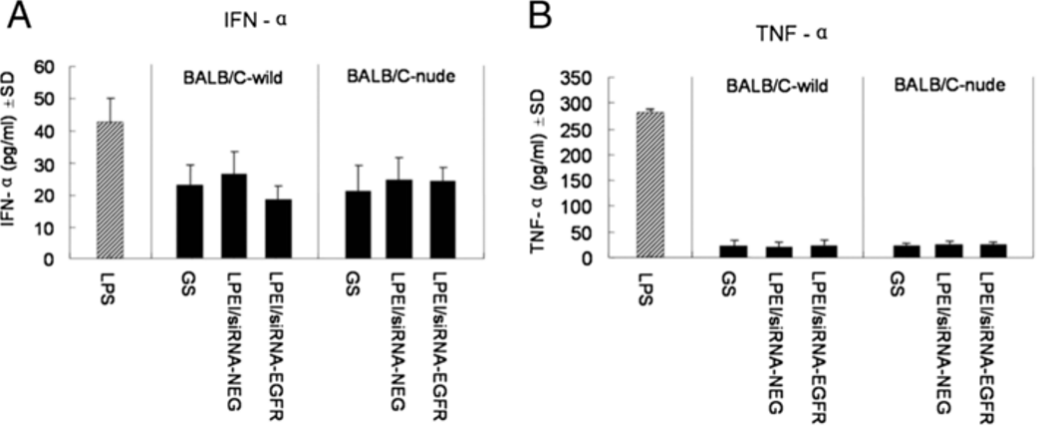
**Serum levels of inflammatory factors.** ELISA assays showing changes in serum levels of IFN-α **(A)** and TNF-α **(B)**. Both cytokines clearly increased after LPS administration, while no significant change was observed in LPEI/siRNA-EGFR, LPEI/siRNA-NEG or GS-administrated groups. All the results are shown as the mean±standard error of the mean. *P < 0.05.

To evaluate the immunogenic reaction following i.p. injection of EGFR-specific siRNA mediated by LPEI, we tested serum levels of pro-inflammatory cytokines, including IFN-α and TNF-α, which have been reported to be increased after siRNA delivered by lipids *in vivo* [32]. Preparation and dose of the LPEI/siRNA complexes were the same as in the above tumor growth inhibition experiment. As indicated in Figure 5A, after the last treatment with 0.6 nmol of LPEI-complexed siRNAs, TNF-α production increases were induced in neither nude nor immunocompetent mice compared with the negative control group. On the other hand, LPS treatment led to a significant increase of serum TNF-α level in the positive control group, as expected. Similarly, at the end of the experiment no significant IFN-α increase was detected in LPEI/siRNA complexes or glucose-treated groups (Figure 5B).

## Discussion

The success of siRNA-based strategies *in vivo* relies on a delivery system which can efficiently protect and carry siRNA across various intracellular and extracelluar barriers into target cells in order to result in sequence-specific mRNA degradation [33]. In light of the poor clinical safety of viral vectors, linear polyethylenimine (LPEI) has emerged as a potent candidate because of its versatility and comparatively high gene transfection efficiency [34]. Because of its potential for DNA delivery, we used a commercial low molecular weight (22 kDa) linear PEI. The *in vitro* data demonstrated that, upon delivery of LPEI, a significant EGFR protein down-regulation was achieved in SPC-A1 cells by transfection of an EGFR-specific siRNA. Its efficiency was comparable to Lipofectamine 2000, a commonly used cationic liposome-based transfection reagent. As reported by many studies [20, 35], this can be explained with the property that cationic LPEI can potently compact negatively charged siRNAs into stable complexes with proper positive surface potential. This will facilitate their interaction with negatively charged cell surface components, and the resulting particle size of less than 100 nm is suitable for endocytosis. This also enhances the complexes’ delivery into cells, eliciting specific RNAi effects [36]. In one study, Urban et al. found that i.p. injection of LPEI-complexed siRNAs (0.8 μg) targeting the c-erbB2/neu (HER-2) receptor resulted in a marked reduction of ovary tumor xenograft growth. [37] Grzelinski used LPEI-complexed siRNA targeting secreted growth factor pleiotrophin (PTN) to treat U87 glioblastoma subcutaneous xenograft-bearing mice As a result, intact siRNA was successfully delivered and a ~40% inhibition of tumor growth was observed over 3 weeks. [27] Furtherly, in the orthotopic glioblastoma mouse model, a low dose (0.2 μg) of LPEI-complexed PTN siRNA was injected into the central nervous system, also exerting an antitumor effect. Similarly, in our present experiment, upon intraperitoneal injection and complexing of LPEI EGFR-specific siRNA (0.8 μg) induced a marked down-regulation of EGFR expression in NSCLC xenografts, also resulting in significant tumor growth inhibition by repeated treatments noted after 3 weeks. There was no statistical difference between the LPEI-complexed non-EGFR-specific siRNA and glucose-treated groups, demonstrating the specificity of target mRNA degradation in mechanism, and no antitumor effect from the non-viral vector was observed. ,Moreover, there are some studies, which reported high dose of naked siRNA delivery in mice by high-pressure tail injections. However, this method was considered to be too toxic and not compatible with clinical application [16]. In comparison, LPEI can carry a lower dose of siRNA to effectively exert an antitumor effect *in vivo* [38].

One key factor for successful RNAi-based therapy is to choose the right target genes. With increasing knowledge of cancer cell signaling pathways, inhibition of critical signal transducers involved in proliferation or survival is a promising direction for developing siRNA-based cancer therapeutics [18]. EGFR is a glycoprotein with a molecular weight of 170,000 to 180,000, and an intrinsic tyrosine-specific protein kinase, stimulated upon epidermal growth factor (EGF) binding. A previous study [31] indicated that significant and specific down-regulation of EGFR expression by vector-based short hairpin RNA (shRNA) can inhibit human lung adenocarcinoma cell (A549 and SPC-A1) growth, induce cell apoptosis, and subsequently increase sensitivity to cisplatin, doxorubicin, and paclitaxel by about 4- to 7-fold in these two HLAC cell lines, respectively. Based on these results, the present study has taken a giant step forward by successfully *in vivo* applying the EGFR-target siRNA complexed with LPEI, eliciting a marked antitumor effect through specific EGFR down-regulation.

Given that it is crucial to identify potential side effects induced by this siRNA-based therapeutic system, toxicity and production of pro-inflammatory cytokines after siRNA-based therapy were examined. As shown in the repeated-injection experiment, no adverse effect on hepatic or renal function was observed from LPEI-complexed siRNA-EGFR treatment. Microscopic examinations of the heart, liver, kidneys and lungs also revealed no detectable damage. As for immunogenicity, treatment with EGFR-specific or non-specific siRNA (0.8 μg, 0.4 mg/Kg body weight) complexed with LPEI did not change serum TNF-α or IFN-α levels compared with glucose-treated mice, which as an indication of a severe immune response, can be triggered by siRNA over-dosage, an improper sequence design, or certain delivery vectors and induce injuries *in vitro* or *in vivo* [32, 39]. Similarly, Grzenlinski found no obvious production of pro-inflammatory cytokines in a glioblastoma mouse model [27], while Bonnet et al. [40] revealed no induction of inflammation or liver damage was observed after i.v. injection of the formulation. Accordingly, the LPEI/siRNA-based therapy seems safe for clinical application.

As a new technology, siRNA-based therapy has rapidly moved into the clinic. Vascular endothelial growth factor (VEGF)-targeted siRNA, bevasiranib, although did not show significant response in the phase III trial, is the first trying towarding siRNA-based tumor targeted therapy. . As, ALN-RSV01, against the mRNA of the respiratory syncytial virus (RSV) nucleocapsid (N) protein, is the first antiviral siRNA to enter clinical trials [41]. Concerning system administration, the first phase I clinical trial of tumor-targeted delivery of siRNA was started in 2008 [42, 43].

The present study has demonstrated that a LPEI-complexed EGFR-specific siRNA delivery system can efficiently and effectively inhibit EGFR expression in SPC-A1 cells *in vitro*. This is the first report of its effective therapeutic application with intraperitoneal injection in a preclinical NSCLC cell xenograft model, with good biological safety.

## Conclusions

Given these results, the novel modality of EGFR-target siRNA-based therapy may have promise in human NSCLC patients.

## Authors’ original submitted files for images

Below are the links to the authors’ original submitted files for images. Authors’ original file for figure 1
Authors’ original file for figure 2
Authors’ original file for figure 3
Authors’ original file for figure 4
Authors’ original file for figure 5

## Abbreviations

EGFR: Epidermal growth factor receptor
NSCLC: Non-small cell lung cancer
RNAi: RNA interference
LPEI: Linear polyethylenimine
i.p.: Intraperitoneal
TKIs: Tyrosine kinase inhibitors
mRNA: Messenger RNA
HIV: Human immunodeficiency virus
HLAC: Human lung adenocarcinoma cell
PBS: Phosphate buffered saline
ELISA: Enzyme-linked immunosorbent assay
shRNA: Short hairpin RNA
EGF: Epidermal growth factor
RSV: Respiratory syncytial virus.

## References

[1] Jemal A, Siegel R, Xu J, Ward E (2010). Cancer statistics, 2010. CA Cancer J Clin.

[2] Carney DN (2002). Lung cancer–time to move on from chemotherapy. N Engl J Med.

[3] Azzoli CG, Temin S, Giaccone G (2012). 2011 Focused Update of 2009 American Society of Clinical Oncology Clinical Practice Guideline Update on Chemotherapy for Stage IV Non-Small-Cell Lung Cancer. J Oncol Pract.

[4] Baldotto CS, Cronemberger EH, De Biasi P, Zamboni M, Sousa A, Zukin M, Small IA, Ferreira CG (2012). Palliative care in poor-performance status small cell lung cancer patients: is there a mandatory role for chemotherapy?. Support Care Cancer.

[5] Booth CM, Shepherd FA, Peng Y, Darling G, Li G, Kong W, Mackillop WJ (2012). Adjuvant chemotherapy for non-small cell lung cancer: practice patterns and outcomes in the general population of Ontario, Canada. J Thorac Oncol.

[6] Ray M, Salgia R, Vokes EE (2009). The role of EGFR inhibition in the treatment of non-small cell lung cancer. Oncologist.

[7] Mok TS, Wu YL, Thongprasert S, Yang CH, Chu DT, Saijo N, Sunpaweravong P, Han B, Margono B, Ichinose Y (2009). Gefitinib or carboplatin-paclitaxel in pulmonary adenocarcinoma. N Engl J Med.

[8] Xu N, Zhang X, Wang X, Ge HY, Wang XY, Garfield D, Yang P, Song YL, Bai CX (2012). FoxM1 mediated resistance to gefitinib in non-small-cell lung cancer cells. Acta Pharmacol Sin.

[9] Engelman JA, Janne PA (2008). Mechanisms of acquired resistance to epidermal growth factor receptor tyrosine kinase inhibitors in non-small cell lung cancer. Clin Cancer Res.

[10] Maheswaran S, Sequist LV, Nagrath S, Ulkus L, Brannigan B, Collura CV, Inserra E, Diederichs S, Iafrate AJ, Bell DW (2008). Detection of mutations in EGFR in circulating lung-cancer cells. N Engl J Med.

[11] Engelman JA, Zejnullahu K, Gale CM, Lifshits E, Gonzales AJ, Shimamura T, Zhao F, Vincent PW, Naumov GN, Bradner JE (2007). PF00299804, an irreversible pan-ERBB inhibitor, is effective in lung cancer models with EGFR and ERBB2 mutations that are resistant to gefitinib. Cancer Res.

[12] Sharma SV, Bell DW, Settleman J, Haber DA (2007). Epidermal growth factor receptor mutations in lung cancer. Nat Rev Cancer.

[13] Burris HA (2009). Shortcomings of current therapies for non-small-cell lung cancer: unmet medical needs. Oncogene.

[14] Edelstein ML, Abedi MR, Wixon J (2007). Gene therapy clinical trials worldwide to 2007–an update. J Gene Med.

[15] Pirollo KF, Chang EH (2008). Targeted delivery of small interfering RNA: approaching effective cancer therapies. Cancer Res.

[16] Lewis DL, Wolff JA (2005). Delivery of siRNA and siRNA expression constructs to adult mammals by hydrodynamic intravascular injection. Methods Enzymol.

[17] Elbashir SM, Harborth J, Lendeckel W, Yalcin A, Weber K, Tuschl T (2001). Duplexes of 21-nucleotide RNAs mediate RNA interference in cultured mammalian cells. Nature.

[18] Kumsta C, Hansen M (2012). C. elegans rrf-1 Mutations Maintain RNAi Efficiency in the Soma in Addition to the Germline. PLoS One.

[19] Liu Q, Muruve DA (2003). Molecular basis of the inflammatory response to adenovirus vectors. Gene Ther.

[20] Lungwitz U, Breunig M, Blunk T, Gopferich A (2005). Polyethylenimine-based non-viral gene delivery systems. Eur J Pharm Biopharm.

[21] Yuan JJ, Jin RH (2011). Approaches to nanostructure control and functionalizations of polymer@silica hybrid nanograss generated by biomimetic silica mineralization on a self-assembled polyamine layer. Beilstein J Nanotechnol.

[22] Hassani Z, Francois JC, Alfama G, Dubois GM, Paris M, Giovannangeli C, Demeneix BA (2007). A hybrid CMV-H1 construct improves efficiency of PEI-delivered shRNA in the mouse brain. Nucleic Acids Res.

[23] Liao HW, Yau KW (2007). In vivo gene delivery in the retina using polyethylenimine. Biotechniques.

[24] Ohana P, Schachter P, Ayesh B, Mizrahi A, Birman T, Schneider T, Matouk I, Ayesh S, Kuppen PJ, de Groot N (2005). Regulatory sequences of H19 and IGF2 genes in DNA-based therapy of colorectal rat liver metastases. J Gene Med.

[25] Wirth M, Fritsche P, Stojanovic N, Brandl M, Jaeckel S, Schmid RM, Saur D, Schneider G (2011). A simple and cost-effective method to transfect small interfering RNAs into pancreatic cancer cell lines using polyethylenimine. Pancreas.

[26] Sidi AA, Ohana P, Benjamin S, Shalev M, Ransom JH, Lamm D, Hochberg A, Leibovitch I (2008). Phase I/II marker lesion study of intravesical BC-819 DNA plasmid in H19 over expressing superficial bladder cancer refractory to bacillus Calmette-Guerin. J Urol.

[27] Grzelinski M, Urban-Klein B, Martens T, Lamszus K, Bakowsky U, Hobel S, Czubayko F, Aigner A (2006). RNA interference-mediated gene silencing of pleiotrophin through polyethylenimine-complexed small interfering RNAs in vivo exerts antitumoral effects in glioblastoma xenografts. Hum Gene Ther.

[28] Akinc A, Thomas M, Klibanov AM, Langer R (2005). Exploring polyethylenimine-mediated DNA transfection and the proton sponge hypothesis. J Gene Med.

[29] Zhang M, Zhang X, Bai CX, Chen J, Wei MQ (2004). Inhibition of epidermal growth factor receptor expression by RNA interference in A549 cells. Acta Pharmacol Sin.

[30] Xu XM, Chen Y, Chen J, Yang S, Gao F, Underhill CB, Creswell K, Zhang L (2003). A peptide with three hyaluronan binding motifs inhibits tumor growth and induces apoptosis. Cancer Res.

[31] Bai L, Zhu R, Chen Z, Gao L, Zhang X, Wang X, Bai C (2006). Potential role of short hairpin RNA targeting epidermal growth factor receptor in growth and sensitivity to drugs of human lung adenocarcinoma cells. Biochem Pharmacol.

[32] Judge AD, Sood V, Shaw JR, Fang D, McClintock K, MacLachlan I (2005). Sequence-dependent stimulation of the mammalian innate immune response by synthetic siRNA. Nat Biotechnol.

[33] Castanotto D, Rossi JJ (2009). The promises and pitfalls of RNA-interference-based therapeutics. Nature.

[34] Billiet L, Gomez JP, Berchel M, Jaffres PA, Le Gall T, Montier T, Bertrand E, Cheradame H, Guegan P, Mevel M (2012). Gene transfer by chemical vectors, and endocytosis routes of polyplexes, lipoplexes and lipopolyplexes in a myoblast cell line. Biomaterials.

[35] Hobel S, Aigner A (2010). Polyethylenimine (PEI)/siRNA-mediated gene knockdown in vitro and in vivo. Methods Mol Biol.

[36] Endres T, Zheng M, Beck-Broichsitter M, Samsonova O, Debus H, Kissel T (2012). Optimising the self-assembly of siRNA loaded PEG-PCL-lPEI nano-carriers employing different preparation techniques. J Control Release.

[37] Urban-Klein B, Werth S, Abuharbeid S, Czubayko F, Aigner A (2005). RNAi-mediated gene-targeting through systemic application of polyethylenimine (PEI)-complexed siRNA in vivo. Gene Ther.

[38] Wang M, Tucker JD, Lu P, Wu B, Cloer C, Lu Q (2012). Tris[2-(acryloyloxy)ethyl]isocyanurate cross-linked low-molecular-weight polyethylenimine as gene delivery carriers in cell culture and dystrophic mdx mice. Bioconjug Chem.

[39] Hornung V, Guenthner-Biller M, Bourquin C, Ablasser A, Schlee M, Uematsu S, Noronha A, Manoharan M, Akira S, de Fougerolles A (2005). Sequence-specific potent induction of IFN-alpha by short interfering RNA in plasmacytoid dendritic cells through TLR7. Nat Med.

[40] Bonnet ME, Erbacher P, Bolcato-Bellemin AL (2008). Systemic delivery of DNA or siRNA mediated by linear polyethylenimine (L-PEI) does not induce an inflammatory response. Pharm Res.

[41] Zamora MR, Budev M, Rolfe M, Gottlieb J, Humar A, Devincenzo J, Vaishnaw A, Cehelsky J, Albert G, Nochur S (2011). RNA interference therapy in lung transplant patients infected with respiratory syncytial virus. Am J Respir Crit Care Med.

[42] Davis ME (2009). The first targeted delivery of siRNA in humans via a self-assembling, cyclodextrin polymer-based nanoparticle: from concept to clinic. Mol Pharm.

[43] Davis ME, Zuckerman JE, Choi CH, Seligson D, Tolcher A, Alabi CA, Yen Y, Heidel JD, Ribas A (2010). Evidence of RNAi in humans from systemically administered siRNA via targeted nanoparticles. Nature.

