# Supplementary figures and images for: A linear polyethylenimine mediated siRNA-based therapy targeting human epidermal growth factor receptor in SPC-A1 xenograft mice
Source: Transl Respir Med. 2013 Feb 22;1:2. doi: 10.1186/2213-0802-1-2 (PMC6733432; doi:10.1186/2213-0802-1-2)

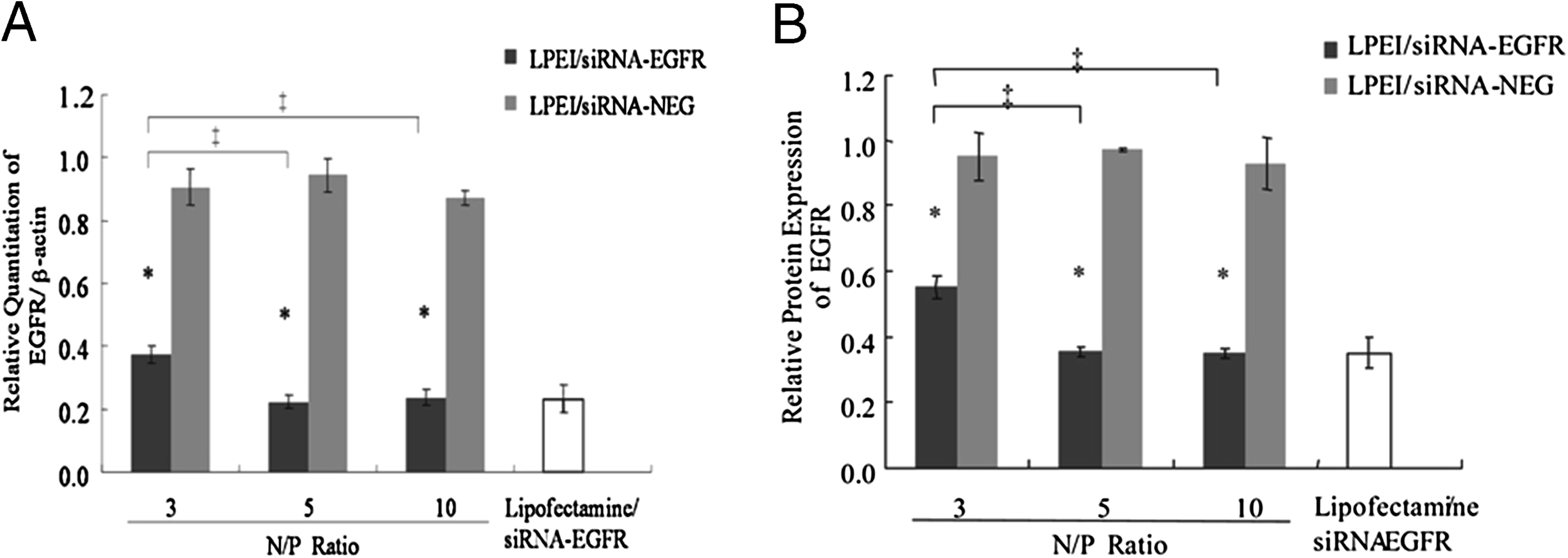

Supplement: Supplementary file 1 — Authors’ original file for figure 1 [file 40247_2012_2_MOESM1_ESM.tiff]

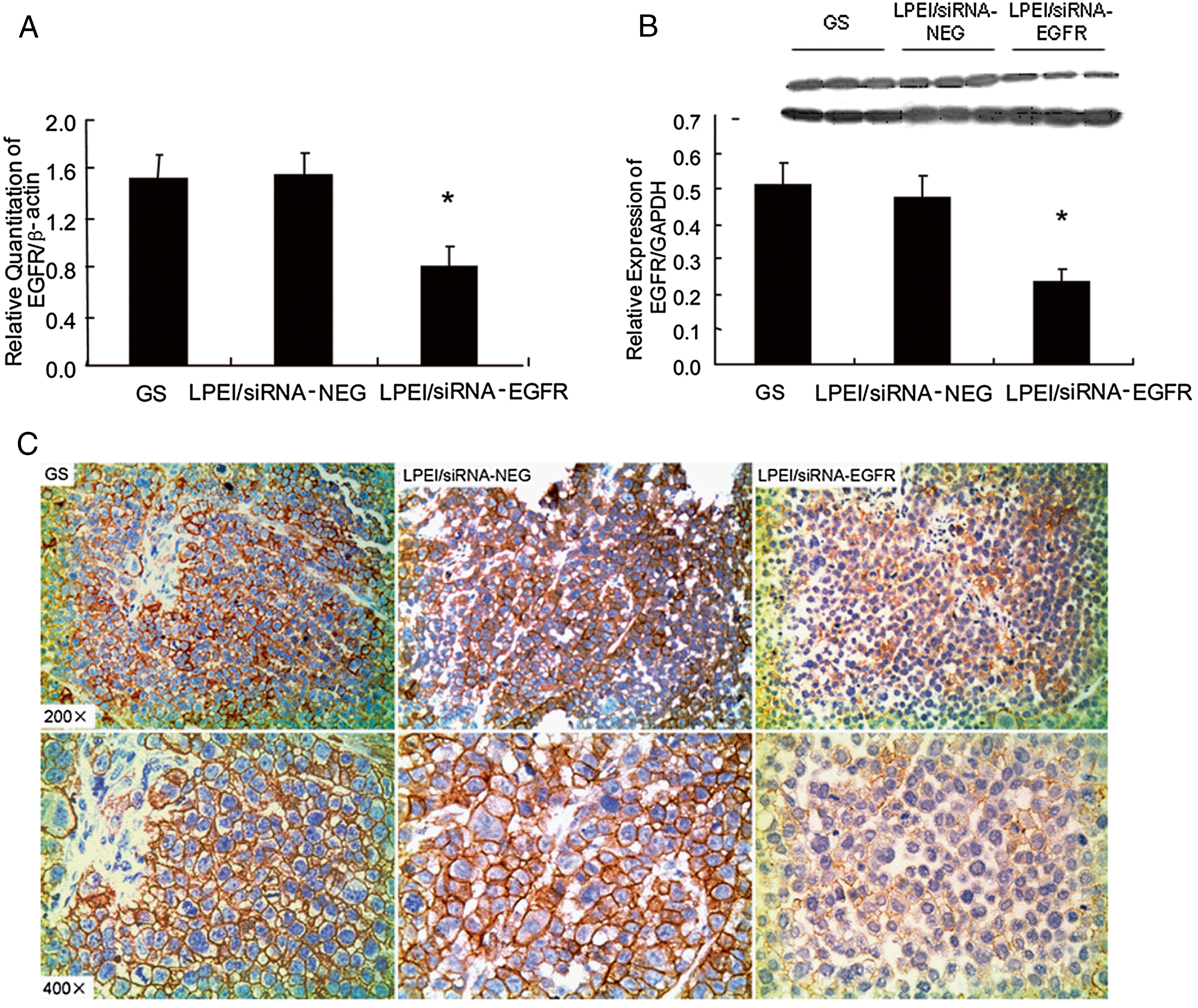

Supplement: Supplementary file 2 — Authors’ original file for figure 2 [file 40247_2012_2_MOESM2_ESM.tiff]

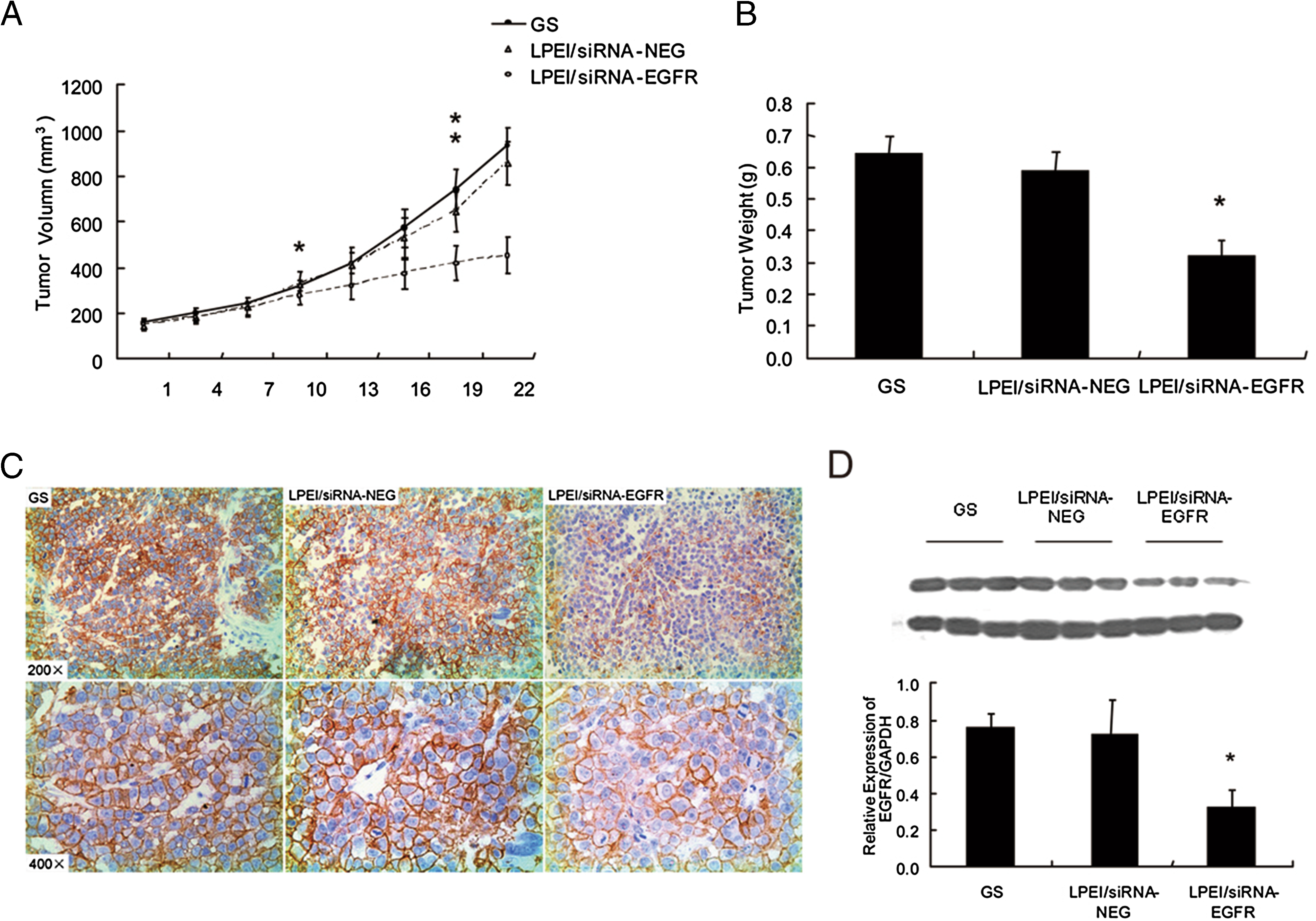

Supplement: Supplementary file 3 — Authors’ original file for figure 3 [file 40247_2012_2_MOESM3_ESM.tiff]

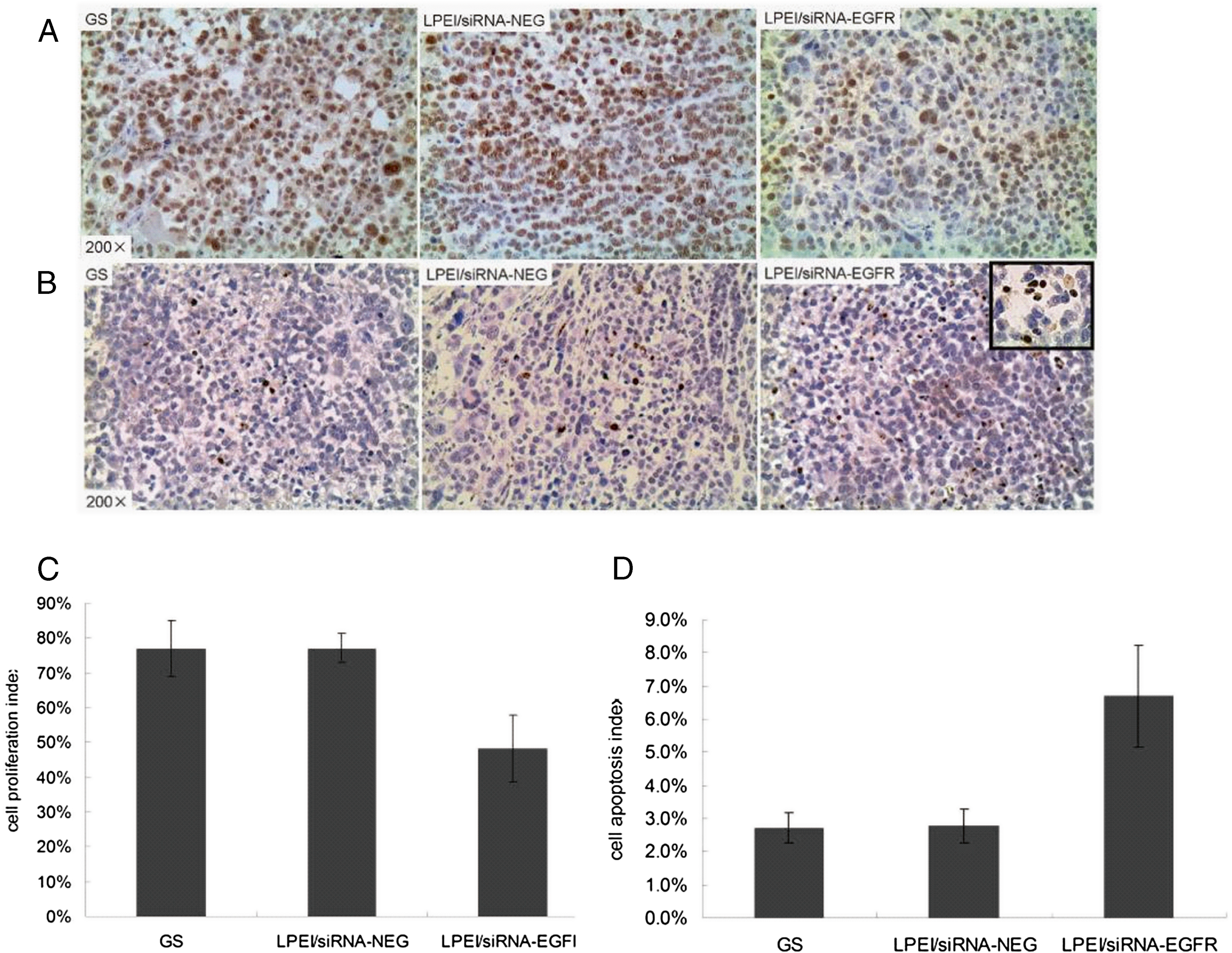

Supplement: Supplementary file 4 — Authors’ original file for figure 4 [file 40247_2012_2_MOESM4_ESM.tiff]

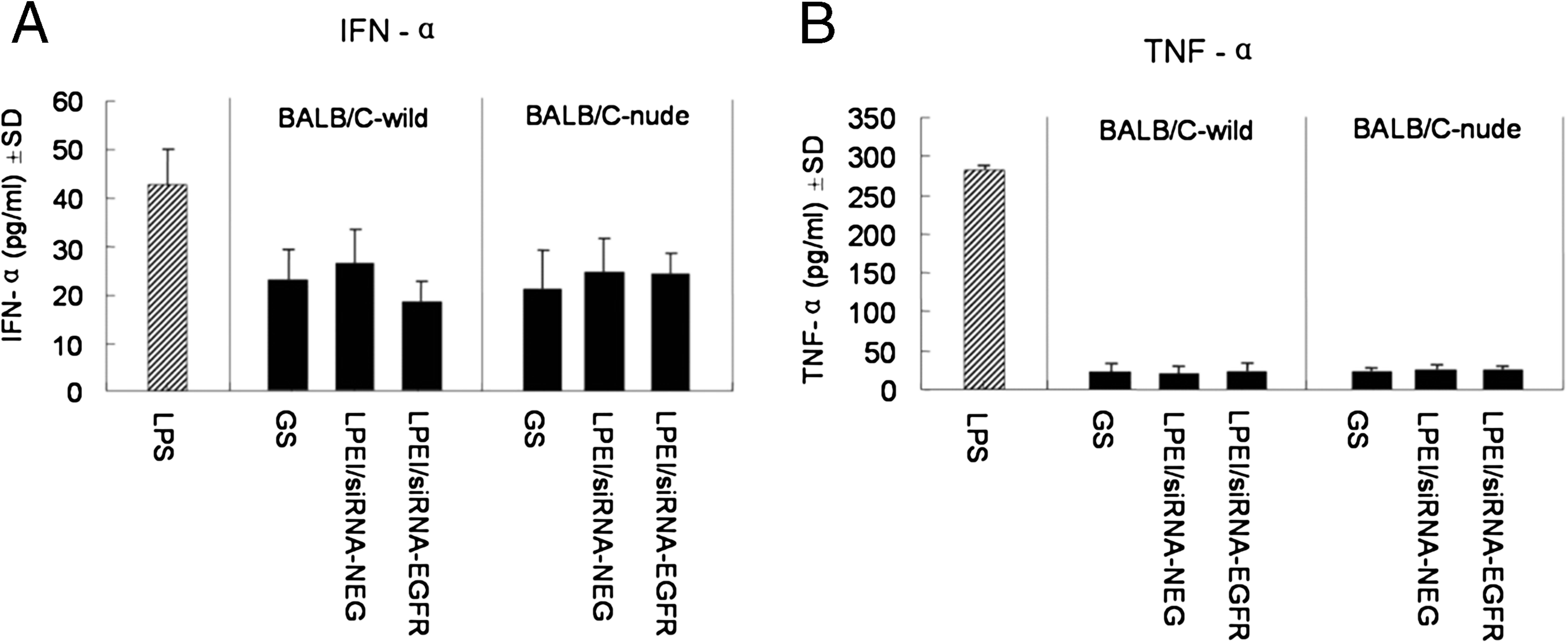

Supplement: Supplementary file 5 — Authors’ original file for figure 5 [file 40247_2012_2_MOESM5_ESM.tiff]
